# Uncontrolled diabetes may cause a transient decrease in ovarian reserve parameters to a suboptimal level: Recovery with optimized glycemic control - A case report

**DOI:** 10.5935/1518-0557.20250156

**Published:** 2025

**Authors:** Mackenzie Ann Campbell, Michael Haim Dahan

**Affiliations:** 1 Department of Obstetrics and Gynaecology, University of British Columbia, Vancouver, British Columbia, Canada V5Z 1M9; 2 Division of Reproductive Endocrinology and Infertility, McGill University Health Centre, Montreal, Quebec, Canada H2L 4M8

**Keywords:** anti-Müllerian hormone, antral follicle count, diminished ovarian reserve, infertility, type 1 diabetes

## Abstract

In this case report, we review a patient in whom ovarian reserve parameters increased significantly following treatment of uncontrolled diabetes. The patient is a previously healthy, lean 29-year-old woman in a same-sex relationship who was seen at a fertility clinic interested in pursuing treatment with donor sperm. Baseline fertility investigations were completed to ascertain the patient’s ovarian reserve and candidacy for intrauterine insemination versus in vitro fertilization. Baseline fertility investigations revealed diminished ovarian reserve putting her at risk for a suboptimal response, with anti-Müllerian hormone level 1.5 ng/mL and antral follicle count 14. Following two unsuccessful cycles of donor sperm intrauterine insemination, the patient presented to the emergency department in diabetic ketoacidosis with hemoglobin A1C 12.3% and random glucose 20.3 mmol/L. She was diagnosed with type 1 diabetes and treated with insulin lispro and bolus insulin. Hemoglobin A1C improved to 6.0% over several months. When she returned to the fertility clinic one year after initial presentation, anti-Müllerian hormone had increased to 7.0 ng/mL and antral follicle count to 44 when performed in the original laboratory and ultrasound unit. We surmise that uncontrolled diabetes may be a cause of spuriously decreased ovarian reserve parameters, which may improve with optimized glycemic control. Further studies are needed to confirm this finding.

## INTRODUCTION

It is estimated that approximately 10% of female patients with infertility have diminished ovarian reserve (DOR) (Levi *et al*., 2001). The definition of DOR remains vague but generally refers to a lower anti-Müllerian hormone (AMH) level or antral follicle count (AFC) than expected compared to others of the same age (Cohen *et al*., 2015). Recently, a publication defined the serum AMH level and baseline AFC putting subjects at risk for a suboptimal ovarian response per the POSEIDON definition. These were a serum AMH level under 2.9 ng/mL and an AFC under 13 (Hochberg *et al*., 2024).

Several factors may lead to DOR including aging, endometriosis surgery, prior cancer treatment, pelvic infections, and environmental exposures (Seyhan *et al*., 2015; Gracia *et al*., 2012; Malhotra *et al*., 2012; Oladipupo *et al*., 2022). Furthermore, endocrine conditions such as type 1 diabetes are also known to negatively affect ovarian reserve. A prospective study in a rat model compared AMH levels between healthy rats and rats with overt diabetes mellitus (Nayki *et al*., 2016). The study team showed significantly higher AMH levels in the healthy rats than in the rats with overt diabetes mellitus. A cross-sectional analysis in human patients has since shown that, when adjusted for covariates, AMH levels were significantly lower in women with type 1 diabetes compared to healthy women under age 35 (Kim *et al*., 2016). A recent retrospective cohort study also showed that infertile female patients with type 2 diabetes have significantly lower AMH levels compared to healthy women, with fewer oocytes retrieved at time of ovum pick-up (Qin *et al*., 2023). However, fluctuations in ovarian reserve parameters due to glycemic control were not described. Though it appears clear that both type 1 and type 2 diabetes are associated with DOR, there is no available literature on the impact of treatment of type 1 diabetes on ovarian reserve testing results.

In this report, we describe a case of a patient with DOR and undiagnosed type 1 diabetes, with significant improvement in ovarian reserve testing results with medical control of type 1 diabetes.

## CASE DESCRIPTION

The patient discussed in this case report provided written informed consent to the use of their clinical data for research purposes.

A previously healthy, lean (BMI 20.2 kg/m^2^) 29-year-old G0 woman was seen in consultation at a fertility clinic in October 2023. She was in a same-sex relationship and was interested in pursuing fertility treatment with donor sperm. Her baseline fertility investigations were normal apart from being non-immune to rubella and having DOR, with AMH level 1.5 ng/mL (modified Beckman Coulter generation II enzyme-linked immunosorbent assay, Beckman Coulter, Canada) and AFC 14. Both fallopian tubes were patent on hysterosalpingogram, so she was deemed a candidate for donor sperm intrauterine insemination (IUI).

The patient underwent one cycle of donor sperm IUI without ovarian stimulation in December 2023 and one cycle of donor sperm IUI with letrozole in February 2024. Pregnancy tests were negative after both cycles.

The patient then presented to the emergency department in May 2024 with malaise. On initial blood work, the patient’s hemoglobin A1C (HbA1C) was 12.3% (reference range <5.0%), random glucose was 20.3 mmol/L (reference range <7.8 mmol/L), and beta-hydroxybutyrate was 0.93 mmol/L (reference range <0.5 mmol/L). Other lab results were as follows: hemoglobin 129 g/L (reference range 120-155 g/L), white blood cell count 6.3 x 10^9^/L (reference range 4.4-10.8 x 10^9^/L), platelet count 230 x 10^9^/L (reference range 150-500 x 10^9^/L), creatinine 55 umol/L (reference range 39-91 umol/L), sodium 134 mmol/L (reference range 134-144 mmol/L), potassium 4.0 mmol/L (reference range 3.6-5.1 mmol/L), chloride 99 mmol/L (reference range 97-108 mmol/L), albumin 45 g/L (reference range 34-46 g/L), magnesium 0.71 mmol/L (reference range 0.74-1.03 mmol/L), TSH 2.18 mU/L (reference range 0.35-6.00 mU/L), and HCG <1 U/L (reference range 0-5 U/L). Her venous blood gases were as follows: pH 7.37 (reference range 7.32-7.42), pCO2 46 mm Hg (reference range 41-51 mm Hg), bicarbonate 26 mmol/L (reference range 24-28 mmol/L), and CO2 total 27 mmol/L (reference range 25-29 mmol/L). All other electrolytes, liver enzymes and urinalysis were normal.

The patient was subsequently admitted to hospital for management of diabetic ketoacidosis and received a new diagnosis of diabetes mellitus. In June 2024, her 65-kD isoform of glutamic acid decarboxylase (GAD65) autoantibody level was >250.0 kUI/L (reference range 0.0-4.9 kUI/L), confirming a diagnosis of type 1 diabetes. The patient was started on insulin lispro and bolus insulin and repeat HbA1C was 6.7% in September 2024. The type 1 diabetes was suspected to be associated with a case of COVID-19, which predated her IUI cycles by more than 8 months.

In December 2024, the patient returned to care at the same fertility clinic, interested in pursuing additional cycles of donor sperm IUI. Baseline fertility investigations were repeated at the same laboratory and ultrasound unit as the year prior. Her type 1 diabetes control was optimized with HbA1C 6.0% on insulin lispro 3-4 units with each meal and insulin degludec 5 units every night at bedtime. Interestingly, her ovarian reserve testing had normalized with repeat AMH level 7.0 ng/mL and AFC 44.

## DISCUSSION

In this case report, we present a patient with a diagnosis of DOR who had a significant improvement in ovarian reserve testing results with medical control of type 1 diabetes. This begs the question whether poor glycemic control is inversely related to ovarian reserve testing results in patients with diabetes.

There is little research on ovarian reserve testing variability and factors that may influence this. However, one factor known to affect ovarian reserve testing results is recent combined oral contraceptive pill use, which is known to temporarily lower AMH levels and AFC (van den Berg *et al.,* 2010). The suggested mechanism for this effect is that the combined oral contraceptive pill use leads to prolonged suppression of follicle stimulating hormone, which inhibits preantral and small antral follicle formation. As a result, the cohort of cells producing AMH is smaller in individuals on combined oral contraceptive pills. Though some studies suggest that this effect may be minimal, a prospective cohort study in 2020 showed that after discontinuation of combined oral contraceptive pills, AMH levels increased by up to 53% (Landersoe *et al*., 2020).

More recently, a study in 2023 suggested that moderate solar radiation exposure may positively impact AMH levels in women aged 30 to 40 years, leading to seasonal variability in AMH testing results (Parikh *et al*., 2023). This may reflect the positive effect of vitamin D on endocrine organ function, including the ovaries (Grzechocinska *et al*., 2013). Otherwise, there are very few studies on the topic of ovarian reserve testing variability, particularly the impact of systemic disease control such as type 1 diabetes.

It would be interesting to explore whether uncontrolled diabetes with poor glycemic control and elevated autoantibody levels affect AMH levels and AFC. If this is found to be the case, it would be important to understand the underlying mechanism, as it is possible that the same effect could be seen in women with other autoimmune conditions.

Furthermore, the possible existence of ovarian reserve testing variability would impact the treatment of infertility by emphasizing disease control as a first priority and may affect the assisted reproductive technologies offered to patients.

## CONCLUSION

Uncontrolled diabetes may be a cause of spuriously decreased ovarian reserve parameters, which may improve with optimized glycemic control. Further studies are needed to confirm this finding.
